# A Scoping Review of Recent Developments in Cellulose-Derived Hydrogels for Dental Applications

**DOI:** 10.3390/pharmaceutics17101252

**Published:** 2025-09-24

**Authors:** Smriti Aryal A C, Md Sofiqul Islam, Marwan Mansoor Mohammed, Lina Abu-Nada, Elaf Akram Abdulhameed, Sangeetha Narasimhan, Snigdha Pattanaik, Ghee Seong Lim

**Affiliations:** 1 Department of Oral and Craniofacial Health Sciences, College of Dental Medicine, University of Sharjah, Sharjah 27272, United Arab Emirates; mmmohammed@sharjah.ac.ae (M.M.M.);; 2 Department of Operative Dentistry, RAK College of Dental Sciences, RAK Medical and Health Sciences University, Ras Al-Khaimah 30248, United Arab Emirates; sofiqul.islam@rakmhsu.ac.ae; 3 Department of Preventive and Restorative Dentistry, College of Dental Medicine, University of Sharjah, Sharjah 27272, United Arab Emirates; 4 Department of Orthodontics, Pediatric and Community Dentistry, College of Dental Medicine, University of Sharjah, Sharjah 27272, United Arab Emirates; 5 Department of Restorative Dentistry, University Malaya, Kuala Lumpur 50603, Malaysia

**Keywords:** bioactive glass, biomaterials innovation, cellulose-based hydrogels, dental application, dental tissue engineering, drug delivery systems, regenerative dentistry

## Abstract

Application of cellulose-based hydrogels in dentistry has gained significant attention. They are emerging as novel biomaterials in the field of tissue engineering, regeneration, and drug delivery in dentistry. The objective of this scoping review is to highlight and summarize recent developments of cellulose-based hydrogels in their designs, reported applications, and laboratory functions. **Methods:** Between the periods of November 2014 and November 2024 (searches completed and datasets locked on 30th Nov 2024), the comprehensive electronic database search was performed in PubMed, Science Direct, Scopus, and MyEBSCO. All the studies that are related to cellulose-based and dentistry were included in this review. This review followed the PRISMA-ScR guidelines for the Preferred Reporting Items for Systematic Reviews and Meta-analysis Extension for Scoping Reviews. **Results:** Out of 518 entries found, 13 studies were qualified for inclusion. When comparative analysis of cellulose-based hydrogel-related studies was performed, most of the included studies were conducted in vitro, and they highlighted significant advancements in their functionality, their inert properties such as mechanical adaptability, design, bioactivity, biodegradability, and clinical potential. **Conclusions:** Cellulose-based hydrogels show great potential in regenerative dentistry, providing a biomimetic platform for tissue regeneration and drug delivery. Addressing present challenges and exploring pathways towards clinical translation will be critical to know their potential in the future. This review critically evaluates the strengths and weaknesses that are used in the current studies and thus, it provides a resource for future research directions for innovations in the field of regenerative dentistry and tissue engineering.

## 1. Introduction

**Figure 1:**
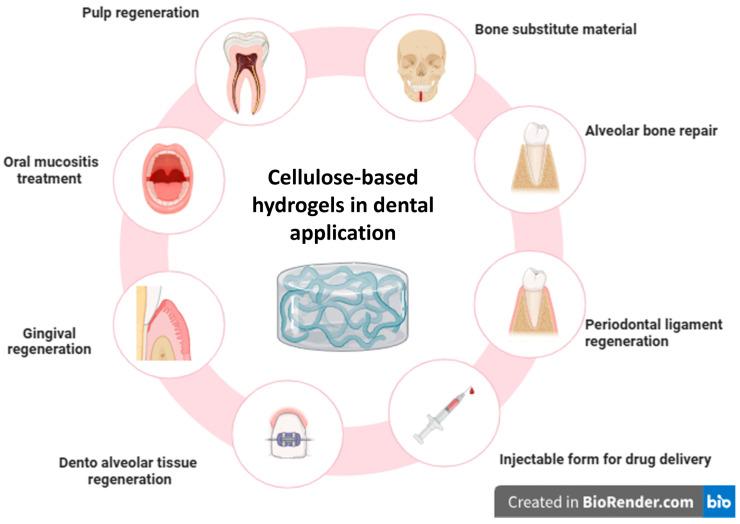
Multifaceted roles of cellulose-based hydrogels in dental applications including dental tissue regeneration, scaffold function, drug delivery, and treatment. The graphical presentation is linked to findings shown in Table 1.

Among human tissues, dental tissues, both soft and hard, are vulnerable to numerous defects and degradation from congenital diseases, trauma, and biological aging [1,2]. In recent years, biomaterials and tissue engineering used for tissue regeneration and repair have significantly increased [3,4,5,6]. Among these materials that were reported, cellulose-based hydrogels have emerged as a promising class of biomaterials for dental applications [7,8]. Hydrogels are three-dimensional hydrophilic polymers that have the capability of absorbing and retaining large amounts of water [9,10]. Their biocompatibility and moldability into various shapes make them suitable for biomedical applications, including tissue engineering and drug delivery [11,12]. In addition to dentistry, the recent developments in hydrogel-based drug delivery systems demonstrate their therapeutic adaptability, controlled release, and responsiveness to physiological stimuli [13]. Beyond therapeutic delivery, recent studies have also highlighted the role of cellulose-based hydrogels in real-time health monitoring, demonstrating their adaptability as multifunctional biomedical tools [14,15]. Moreover, biomedical materials science research has shown that cellulose-derived composites can be manufactured with tunable porosity, bioactivity, and mechanical stability for regenerative application [16]. Significant mechanical and biological changes occur in tooth tissues, including dentin and pulp, demanding the use of a scaffold that closely replicates the native extracellular matrix while also promoting regeneration. This creates the perfect environment for mineralization, differentiation, and cell proliferation [17]. In recent days in the field of regenerative dentistry, cellulose and its derivatives are gaining popularity with tremendous potential in dental tissue engineering as bioactive materials and for drug delivery because of its innate properties such as biocompatibility, mechanical adaptability, and biodegradability, as shown in Figure 1 [18,19].

Cellulose is a polysaccharide that is derived from the cell walls of the plant and has a unique molecular structure that allows for creating hydrogels that have the capability to sustain a moist environment, demonstrate strong mechanical strength, and their adaptable properties, which make them an ideal candidate to be used as a scaffold in dental applications [20]. Derivates of cellulose such as cellulose acetate, oxidized pullulan, gelatin composites, nanocellulose, carboxymethyl cellulose, and bacterial cellulose have been the focus of recent research. All of these derivatives can be engineered into hydrogels, which have shown properties that provide mechanical support for biological processes such as the attachment of cells and their proliferation and differentiation, which are particularly needed for applications in regenerative dentistry [18,21,22]. By adding bioactive glass nanoparticles (BG-NPs) and functional ions like boron (B), which have demonstrated encouraging outcomes in promoting odontogenic and osteogenic differentiation, recent developments have further improved the functionality of cellulose-based hydrogels. For example, it has been demonstrated that boron-modified bioactive glasses greatly enhance calcium phosphate deposition, a crucial stage in dentin regeneration. These bioactive hydrogels are especially well-suited for endodontic applications where structural integrity is crucial because they also have enhanced mechanical qualities and regulated degradation profiles [23,24]. Dental pulp stem cells (DPSCs) have created new avenues for customized regeneration treatments through their incorporation into cellulose-based hydrogels. When paired with bioactive hydrogels, DPSCs’ strong proliferation ability and differentiation potential produce constructions that closely resemble the dentin–pulp complex found in nature. Research has demonstrated that cellulose-based hydrogels can promote the odontogenic differentiation of DPSCs by upregulating the expression of markers such as dentin sialo phosphoprotein (DSPP), osteopontin (OPN), and alkaline phosphatase (ALP) [24,25,26,27,28,29]. Despite the tremendous advancements, optimizing the characteristics of cellulose-based hydrogels for clinical translation remains difficult; thus, it is necessary to solve issues including obtaining ideal porosity, guaranteeing uniform nanoparticle distribution, and striking a balance between tissue development and degradation rates. Furthermore, more research is necessary to improve the clinical effectiveness of hydrogels by better understanding how they interact with biological systems in dynamic oral environments. The results of the most recent peer-reviewed literature are combined in this systematic scoping review to offer a thorough examination of the developments in cellulose-based hydrogels for dental applications. This review summarizes recent laboratory research on cellulose-based hydrogels, focusing on design, mechanical characteristics, bioactivity, and integration with cell therapies. Although the lack of in vivo validation still limits their translation into clinical dentistry, the information that has been gathered here may be of great value to the researchers and clinicians trying to develop next-generation biomaterials for regenerative dentistry as it will provide resources for guiding future research directions in regenerative dentistry and tissue engineering.

## 2. Materials and Methods

### 2.1. Protocol Registration and Research Question

**Figure 2:**
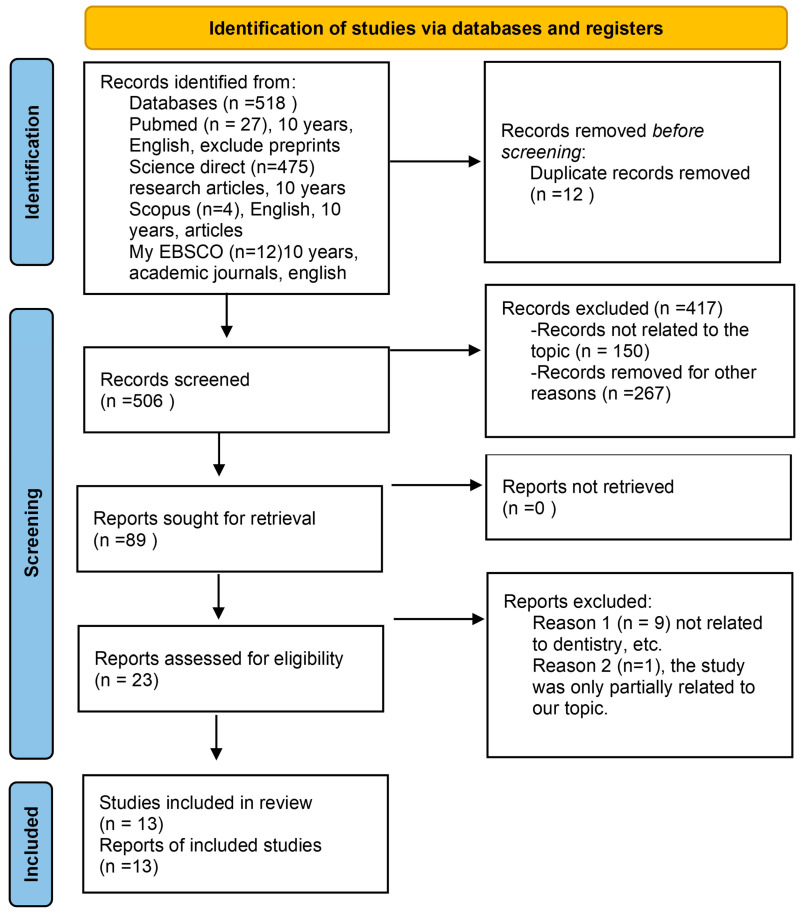
PRISMA-ScR flow diagram used for study selection.

The Preferred Reporting Items for Systematic Reviews and Meta-analyses (PRISMA, Supplementary Materials S1) guideline [30] was followed in the preparation of this review, as shown in Figure 2. The scoping review process has been registered in the Open Science Framework (https://doi.org/10.17605/OSF.IO/D8VT5 (16 September 2025)). “In dental applications, how do modified cellulose-hydrogels help promote dental tissue regeneration, improve drug delivery, and enhance adhesion?” was the focused research question used in this study using the PICO framework, where (P) the population is in dental applications, intervention (I) is modified cellulose–hydrogels, comparison (C) is other dental materials, and outcome (O) is promoting dental tissue regeneration, improving drug delivery, and enhancing adhesion.

### 2.2. Eligibility Criteria

#### 2.2.1. Inclusion Criteria

Inclusion criteria for this scoping review involved the following: (a) research articles published within the last 10 years between November 2014 and November 2024 (searches completed on 30 November 2024 and datasets locked), (b) articles published in English language, (c) full-text research articles published in academic journals, and (d) studies related to cellulose-based hydrogels that are used in regeneration in dentistry or dental applications such as dental tissue regeneration, drug delivery, mucoadhesives, etc.

#### 2.2.2. Exclusion Criteria

Exclusion criteria for this scoping review involved the following: (a) articles published more than 10 years ago or before November 2014, (b) articles published were not written in English language, (c) autobiography, bibliography, biography, books and documents, interview, lecture, legal case, legislation, letter, meta-analysis, news, newspaper, article, preprint, review, scientific integrity review, or systematic review, (d) studies that are related to cellulose-based hydrogels and tissue regeneration but not related to dentistry or dental applications, only partially related and lacking experimental evidence, and (e) articles not published in academic journals.

### 2.3. Data Sources and Search Strategy

During the period between November 2014 and November 2024, the initial searches for this study were systematically performed by the means of four electronic databases, which are PubMed, Science Direct, Scopus, and MyEBSCO. We used these databases as they are recognized for the high-quality, peer-reviewed literature related to medicine and dentistry. Only English-language publications published during this period were selected. The keywords or terms used for the search were the following: for PubMed, (cellulose [All Fields] AND hydrogels [All Fields] OR hydrogels [MeSH Terms]) AND (regeneration [MeSH Terms] OR regeneration [All Fields]) AND (dentistry [MeSH Terms] OR dental [MeSH Terms] OR dental [All Fields]) AND (application [All Fields] OR “dental application” [All Fields]). For Science Direct, Scopus, and MyEBSCO, the terms used for the search were the following: (cellulose AND hydrogels) AND (regeneration) AND (dentistry OR dental) AND (application OR dental application). Studies not in English, review articles, or preprints were all excluded.

### 2.4. Choosing the Sources of Evidence

Following a search of the designated databases, the identified articles underwent different stages of a screening process in accordance with the eligibility criteria. The retrieved articles were initially exported to the Rayyan^®^ online application where, by using the application, duplicate articles were removed. Then, the screening process of the literature was carried out using the Rayyan QCRI web tool [31], which allowed independent access to retrieved articles, allowing each author to work independently (S.A.A.C., M.S.I, and M.A.M). In the initial stage of screening, the title and the abstract were screened to detect the eligible articles. The articles that did not fulfill the inclusion criteria were all excluded. In the second stage of screening, the full text of the selected articles was screened to decide the studies that were suitable for inclusion.

### 2.5. Data Items and Data Charting Process

Two researchers (S.A.A.C. and M.M.M) developed and improved the charting table. Independent data extraction was performed, which includes the following: study id containing authors’ name and year of publication, study type, material/scaffold composition, objective, study medium, methods of analysis, outcomes, dental applications, and limitations of the study.

### 2.6. Critical Appraisal of Each Evidence Source

According to Trico et al., 2018, the risk-of-bias evaluation is optional and is not part of the scoping review process [30].

### 2.7. Results Synthesis

The findings were narratively and qualitatively presented, classified, and summarized.

## 3. Results

### 3.1. Study Selection and Data Charting

The authors independently identified the literature by searching the databases specified above as well as other sources. The process is detailed in the PRISMA flowchart, as shown in Figure 2, and the search was conducted as follows: Identification phase: 518 articles were extracted from the databases in total. Articles that were retrieved were exported to the Rayyan web application. After comparing the titles, 12 were removed for duplicate records, ending with 506 articles in this phase. Screening phase: The literature screening process was carried out by using the Rayyan qcri tool [31]. By screening the title and abstracts of the articles, 417 were excluded and then 89 were selected for retrieval. Then, after further screening of the full text of the articles, a total of 23 articles were selected. Eligibility phase: Out of 23 articles, the final 13 articles were included. The rest of the 10 articles were excluded because they were only partially related to the topic or not totally related to dentistry. Inclusion phase: 13 studies were included in the qualitative synthesis and reported in this review.

### 3.2. Features of the Source of Evidence

Thirteen articles were reviewed, most of which were experimental investigations into the use of hydrogels based on cellulose in dental applications. These resources mostly concentrate on creating and describing scaffolds that mix boron-modified bioactive glass nanoparticles (B-BG-NPs) with cellulose derivatives such as cellulose acetate, pullulan, and gelatin [24,26,32,33,34]. In order to mimic the tubular structure of dentin, the scaffolds were created utilizing porogen leaching and thermally induced phase separation procedures. Robust procedures, such as in vitro degradation tests, porosity measures, mechanical property evaluations, and bioactivity assessments [24,26,34], define the evidence foundation. Scaffold shape and cellular interactions were thoroughly revealed by means of sophisticated imaging and analysis methods such as confocal laser scanning microscopy, energy-dispersive X-ray spectroscopy (EDX), and scanning electron microscopy (SEM) [24,32,35]. Additionally, to better understand the role of these materials in dental tissue engineering, mechanical, biological, and structural evaluations were performed [24,26,32,33,34,35]. The included study included studies where in vitro experiments were performed using pre-osteoblast cell line (MC3T3-E1), human dental pulp stem cells (hDPSCs), and human gingival fibroblast cells. These cells were loaded on the cellulose-based hydrogel scaffold and cell adhesion, proliferation, odontogenic, or osteoblastic differentiation and regenerative capacity were evaluated. Furthermore, the studies also evaluated the scaffolds’ water absorption capacity, porosity, and biodegradation capability. The hydrogels that were used for dental tissue engineering were often combined with gelatin, oxidized pullulan, and cellulose acetate. Scaffolds devoid of bioactive compounds or standard polymeric and ceramic materials are frequently used as control groups for comparison. The data demonstrate how cellulose-based hydrogels have a great deal of potential for simulating the extracellular matrix and promoting dental tissue regeneration (Table 1, Table A1 and Table A2).

**Table 1:** Characteristics and main findings of the included studies.

Author (s), Year	Study Type	Material/ Scaffold Composition	Objective	Study Medium	Methods of Analysis	Outcomes	Dental Applications	Limitations
**Rad et al., 2019** [24]	In vitro	CAox-PULLGEL ^1^ + BG-NPs ^2^	Dentin regeneration	hDPSCs ^3^	SEM ^4^ Cell culture assays	↑ ^5^ Cell adhesion, proliferation, ALP ^6^ activity, calcium deposition, odontogenic differentiation	Regenerative endodontics	In vitro
**Divband et al., 2021** [26]	In vitro	Chitosan, biguanidine, CMC ^7^ + VEGF ^8^ and BMP-2 ^9^	Osteogenic development of hDPSCs	hDPSCs	qRT-PCR ^10^, Western blot MTT ^11^ assay	↑ Proliferation and osteogenic differentiation	Regenerative endodontics	In vitro
**Malik et al., 2020** [32]	In vitro	Thyroxine-loaded chitosan/CMC-HA ^12^	Periodontal regeneration via angiogenesis	CAM ^13^-MC3T3-E1 ^14^	SEM ^15^ FTIR ^16^ Cytotoxicity assays CAM ^17^ assay	↑ Angiogenic activity, non-toxic to cells	Periodontal regeneration	In vitro
**Huerta et al., 2020** [33]	In vitro	CNF ^18^ scaffolds (HIUS ^19^ processed)	Cytocompatibility for periodontal therapy	Human gingival fibroblasts	Cell viability and proliferation assay	↑ Cell viability, proliferation	Periodontal therapy	In vitro
**Amoli et al., 2024** [34]	In vitro	pNIPAM ^20^-methylcellulose microgels	Drug delivery in dentoalveolar engineering	hDPSCs	Hydrodynamic size, VPTT ^21^, drug loading and cytocompatibility	Effective copolymerization, drug delivery	Drug delivery in dento-alveolar tissue	In vitro
**Zeeshan et al., 2018** [35]	In vitro	CH ^22^-HPMC ^23^ + BG ^24^ + ZNO ^25^	Alveolar bone repair	MC3T3-E1	SEM FTIR Mechanical tests Cell viability assays	Good biocompatibility, ↑ osteoblast differentiation	Alveolar bone repair	In vitro
**Pagano et al., 2019** [36]	In vitro and ex vivo	NaCMC ^26^ + CH ^27^ thermosensitive gel	Oral mucositis and drug delivery	Porcine mucosa	Rheological test, ex vivo mucoadhesion Drug release	↑ Mucoadhesion, sustained drug release	Oral mucositis	Limited polymer range No human testing
**Lu et al., 2021** [37]	In vitro and in vivo (rats)	COF-HEC ^28^ iodine hydrogel	Sustained iodine release for periodontitis	Rat periodontal cells, artificial saliva	TGA ^29^ Titration Molecular docking	Sustained iodine release ↓ ^30^ bone resorption in vivo Comparable efficacy to minocycline ointment in treating periodontitis.	Periodontitis	Rat model—not performed on human
**Liu et al., 2014** [38]	In vitro	Si-HPMC ^31^ hydrogel composite CPC ^32^	Mechanical property evaluation	No cells	Injectability, compressive strength, SEM, XRD ^33^, FTIR.	↑ Cohesion, mechanical strength, porosity	Bone substitute material for clinical application	Air bubble entrapment Delayed CDHA ^34^ formation
**Dalir et al., 2022** [27]	In vitro	CS ^35^ + OCNCs ^36^ + MTA ^37^ hydrogel	Injectable regenerative scaffold	hDPSCs	Gelation time FTIR SEM MTT assay ALP activity ARS ^38^ staining	↑ ALP activity, differentiation, proliferation, and functional calcium nodules	Regenerative endodontic	In vitro and short-term study
**Teti et al., 2015** [25]	In vitro	CMC-HA ^39^ hydrogel	Osteogenic/odontogenic differentiation of DPSCs	hDPSCs	MTT assay RTPCR ^40^ EM ^41^ analysis	↑ Biocompatibility, cell adhesion, osteogenic, and odontogenic differentiation	Dental pulp/periodontal regeneration	Small sample size No in vivo validation
**Tritean et al., 2024** [39]	In vitro	CSFa ^42^ + BNC ^43^ + SeNPsK ^44^ hydrogel	-Biological and mechanical evaluation	HGF-1 ^45^	DLS ^46^ ELISA ^47^ Antimicrobial assays	↑ Anti-inflammatory, antimicrobial, and antioxidant activity	Dental implants, periodontal	No in vivo study
**Srisura et al., 2024** [40]	In vitro	CMC + CaP ^48^ + MeHA ^49^ hydrogel	Injectable scaffold for bone regeneration	MC3T3-E1	DLS FTIR NMR ^50^ TEM ^51^ and SEM	↑ Stability, biocompatibility ↑ Injectability ↑ Osteoblast proliferation and mineralization	Injectable scaffold for bone regeneration	No in vivo testing

1. CAox-PULLGEL—cellulose acetate-oxidized pullulan–gelatin; 2. BG-NPs—boron-modified bioactive glass nanoparticles; 3. hDPSCs—human dental pulp stem cells; 4. SEM—scanning electron microscopy; 5. ↑—increased; 6. ALP—alkaline phosphatase; 7. CMC—carboxymethyl cellulose; 8. VEGF—vascular endothelial growth factor; 9. BMP 2—bone morphogenic protein 2; 10. qRT-PCR—quantitative real-time polymerize chain reaction; 11. MTT—3-(4, 5-dimethylthiazol-2-yl)-2,5-diphenyltetrazolium bromide; 12. CMC-HA—carboxymethyl cellulose–hydroxyapatite; 13. CAM—chick chorioallantoic membrane; 14. MC3T3-E1—mouse osteoblast cell line; 15. SEM—scanning electron microscopy; 16. FTIR—Fourier transform infrared spectroscopy; 17. CAM—chick chorioallantoic membrane; 18. CNF—cellulose nanofibers; 19. HIUS—high-intensity ultrasound; 20. pNIPAM—poly(*N*-isopropylacrylamide); 21. VPTT—volume phase transition temperature; 22. CH—chitosan; 23. HPMC—hydroxypropyl methylcellulose; 24. BG—bioactive glass; 25. ZNO—zinc oxide; 26. NaCMC—sodium carboxymethyl cellulose; 27. CH—chitosan; 28. COF-HEC—crosslinked cyclodextrin metal–organic framework suspended in hydroxyethyl cellulose gel; 29. TGA—thermogravimetric analysis; 30. ↓—-decreased; 31. Si-HPMC—silanized-hydroxypropyl methylcellulose; 32. CPC—calcium phosphate cement; 33. XRD—X-ray diffraction; 34. CDHA—calcium-deficient hydroxyapatite; 35. CS—chitosan; 36. OCNC—oxidized-cellulose nanocrystals; 37. MTA—mineral trioxide aggregate; 38. ARS staining—Alizarin Red S staining; 39. CMC-HA—carboxymethyl cellulose-hydroxyapatite; 40. RTPCR—reverse transcription polymerase chain reaction; 41. EM—electron microscopy; 42. CSFa—ferulic acid-grafted chitosan; 43. BNC—bacterial nanocellulose; 44. SeNPsK—selenium nanoparticles from Kombucha fermentation; 45. HGF-1—human gingival fibroblast; 46. DLS—dynamic light scattering; 47. ELISA—enzyme-linked immunoassay; 48. CaP—amorphous calcium phosphate; 49. MeHA—methacrylated hyaluronic acid; 50. NMR—nuclear magnetic resonance; 51. TEM—transmission electron microscopy.

### 3.3. Results of Outcomes

#### 3.3.1. Primary Outcomes

**Figure 3:**
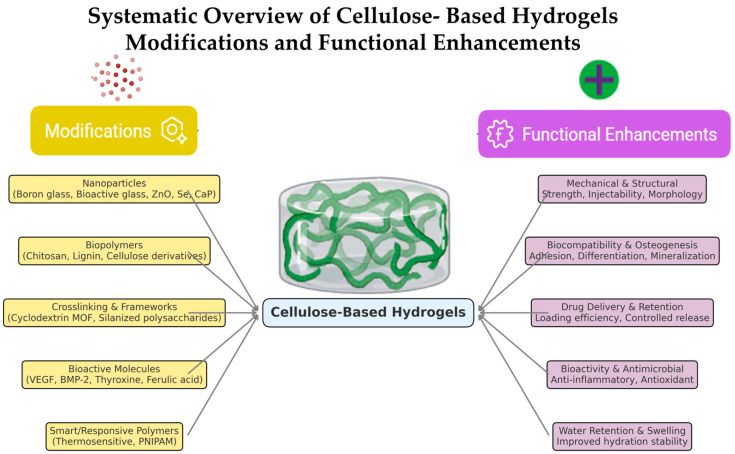
Modifications of cellulose-based hydrogels that enhance different types of functional properties (e.g., degradation, bioactivity, porosity), based on the included studies.

Overall, the consistent finding observed across all the studies was the controlled biodegradation of cellulose-based hydrogels. Degradation rates were greatly affected by the addition of the B-BG-NPs to the scaffolds; over time, the weight loss reduced in scaffolds modified with boron. The best degradation profile was shown by scaffolds containing 10% boron-modified bioactive glass (B14-10), which balanced structural stability with slow disintegration to promote tissue regeneration; however, compared to the scaffolds containing higher (20% nanoparticle concentrations) scaffolds, those which contained to the 10% boron-modified bioactive glass nanoparticles (B-BG-NPs) were found to degrade gradually, showing consistent weight loss throughout the period of 30 days. Furthermore, the degradation rate of the hydrogels was improved with the addition of oxidized cellulose nanocrystals (OCNCs), which were also useful in regenerating the pulp–dentin complex, thus helping in regenerative dental applications. In some included studies, chitosan and other bioactive ingredients (hydroxyapatite) were added to control the degradation rates of the scaffold. The addition of these ingredients not only controlled the degradation rate but also helped the scaffold to preserve the structural stability, as shown in Figure 3.

In addition to the degradation rate and structural stability, the included studies also showed that hDPSCs that were cultured on these scaffolds could differentiate into odontogenic lineage; these results were validated by an increase in alkaline phosphatase (ALP) activity, significant deposition of the mineralized matrix as well as increased accumulation of intracellular calcium levels. In addition, immunohistochemical analysis supported these findings by showing that the boron-modified scaffold groups had higher levels of expression of dentin sialo phosphoprotein (DSPP) and other dentin markers [24,26,32,33,34,35]. Calcium phosphate deposition and hydroxycarbonate apatite formation, which are essential for dentin regeneration, were also enhanced by hydrogels containing boron-modified bioactive glass nanoparticles (B-BG-NPs). Incorporation of oxidized cellulose nanocrystals (OCNCs) also improved the hydrogel’s degradation behavior, aligning with the regenerative requirements of dental applications like pulp–dentin regeneration [25,27,36,37,38,39].

#### 3.3.2. Secondary Outcomes

Mechanical properties, water absorption, and scaffold porosity were the main topics of the secondary outcomes. Scaffolds containing B-BG-NPs showed increased water absorption capacity and porosity, allowing an ideal environment for the cell infiltration and diffusion of nutrition. Scaffold porosity closely resembled that of natural dentin with pores interconnected that facilitated vascularization and tissue integration. Mechanically, at optimal nanoparticle concentrations, boron-modified scaffolds had higher compressive strengths and modulus of elasticity. The best mechanical properties were provided by the B14-10 group, indicating strong interfacial interaction between the polymer matrix and the inorganic nanoparticles. Nevertheless, a high BG-NP content, which is 20%, decreased the mechanical properties due to the disruptions of the matrix. Nanoparticle-treated hydrogels, such as B-BG-NPs, demonstrated enhanced elastic modulus and compressive strength (up to 0.40 MPa), which qualified them for use in dental applications. Performance was greatly affected by filler concentrations that were too high (20%). Hydrophilic nanoparticles enabled greater water absorption, which ensured hydration and improved cell viability for oral tissue engineering [25,27,36,37,38,39,40]. The combination of mineral trioxide aggregate (MTA) with oxidized cellulose nanocrystals (OCNCs) also increased mechanical stability and produced a strong framework for endodontic applications. The cellulose-based hydrogels’ porosity levels, which varied from 91% to 94%, allowed adequate fluid exchange and promoted cellular penetration, which was good in simulating natural dentinal tubules that were tubular constructions with hole sizes of around 11 μm [25,27,36,37,38,39,40].

#### 3.3.3. Tertiary Outcomes

Regarding the tertiary outcomes, the bioactivity, antimicrobial, and antioxidant properties and potential of cellulose-based hydrogels for drug delivery were reported. The addition of boron-enhanced bioactive glass nanoparticles induced the production of apatite layers on scaffold surfaces, resembling the mineralized matrix found in natural dentin. Cell proliferation and differentiation were further promoted by the sustained release of bioactive ions, such as calcium and boron. Confocal imaging and SEM demonstrated that hDPSCs attached well to the surfaces of the scaffold, spreading widely and forming cellular extensions, indicating active odontogenic processes [24,26,32,33,34,35]. Certain formulations, such as bacterial nanocellulose hydrogels enriched with selenium, have significant antibacterial and antioxidant capabilities, making them suitable for use in dental implants and periodontal therapy. Using hydrogels made from cellulose, in situ gelling formulations for the treatment of oral mucositis demonstrated effective localized drug release. Benzydamine hydrochloride, which is one of the ingredients of the medication, was released from the hydrogels in a controlled manner, thus protecting the mucosal surface physically by using the optimized formulations [25,27,36,37,38,39,40].

### 3.4. Synthesis of Results

In total, out of 13 studies, 12 were in vitro and 1 in vivo. The cellulose derivatives most frequently used were cellulose acetate, carboxymethyl cellulose, hydroxyethyl cellulose, hydroxypropyl methylcellulose, bacterial nanocellulose, and oxidized cellulose nanocrystals, often combined with bioactive glass nanoparticles. Reported compressive strengths ranged from ~0.27 to 0.45 MPa, with higher values at optimal filler loading and decreases at excessive concentrations. Porosity values were consistently high (~91–94%), supporting cell infiltration, and 28-day degradation rates ranged from ~7 to 37% depending on composition. Cell viability assays generally showed non-toxicity and enhanced proliferation, although several studies did not provide exact percentages. These pooled outcomes are summarized in Table A1. The synthesis of the findings of the studies highlights the importance of cellulose-based hydrogels as advanced materials for dental tissue engineering. The incorporation of boron-modified bioactive glass nanoparticles improved scaffold performance, resulting in increased bioactivity, customized biodegradability, and excellent mechanical qualities. The B14-10 group proved to be a consistently high-performing scaffold, particularly in terms of hDPSC differentiation and mineralized matrix deposition. This evidence indicates that cellulose-based hydrogels are promising candidates for use in regenerative endodontics and dentin regeneration. Additionally, cellulose-based hydrogels are not only used as a scaffold but have also shown great potential as an antibacterial as well as in drug delivery applications [25,27,36,37,38,39,40]. Thus, studies in the future should focus more on turning the in vitro findings into therapeutic applications in in vivo settings. Furthermore, creating scaffold designs based on the requirements of each patient may also enhance their therapeutic potential in dental applications [24,26,32,33,34,35].

## 4. Discussion

**Figure 4:**
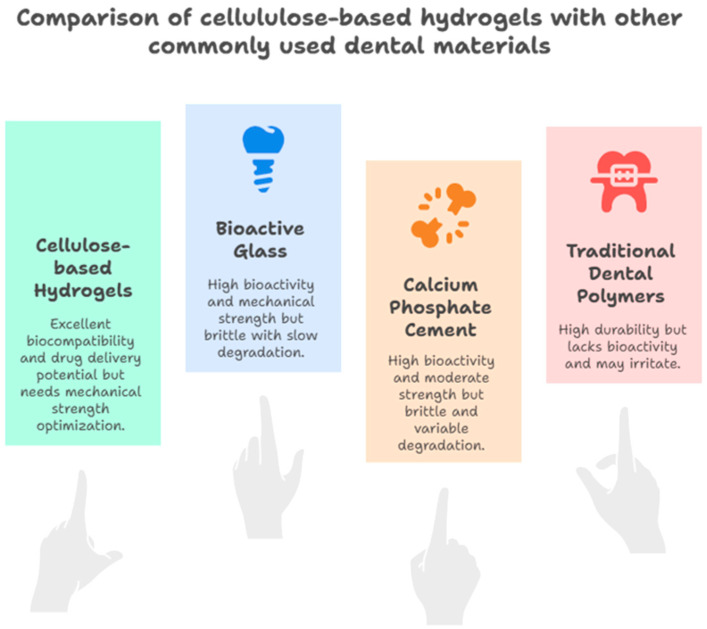
Comparison of cellulose-based hydrogels with other commonly used dental materials, highlighting differences in biocompatibility, drug release, and regenerative potential.

In this scoping review, we highlighted recent laboratory developments using cellulose-based hydrogels for potential dental applications including wound healing, tissue engineering, and drug delivery. While it is promising, however, most evidence remains at the pre-clinical stage and is primarily based on in vitro studies and thus requires more cautious interpretation [41,42]. Although numerous types of hydrogels have been studied, among them, cellulose-based hydrogels, because of their capability to encapsulate and release drugs, growth hormones, or antibiotics at the targeted treatment sites such as wounds, dental infections, and dental diseases, stand out to be unique [43], as shown in Figure 4.

Another development in this field is the generation of injectable cellulose-based hydrogels that can be used for less invasive dental applications; additionally, these hydrogels can be directly applied to the defective areas, allowing the formation of a stable structure and bioactive chemicals or cells that can be incorporated into it for targeted repair or regeneration [44,45,46]. The design of cellulose-based hydrogels for dental applications has also been completely transformed by the use of cutting-edge production techniques like microfluidics and 3D printing. By using these technologies, the architecture of the hydrogels can be precisely controlled, resulting in the production of the special type of scaffolds that show ideal mechanical characteristics. Thus, these developments are very important for improving the functionality of these materials by simulating the internal microenvironment of tooth tissues [47].

This scoping review analyzed the potential applications of cellulose-based hydrogels in oral tissue regeneration and their potential for dental applications. According to the findings of the included studies, cellulose-based hydrogels are biocompatible, they encourage cellular adhesion and proliferation, and these hydrogels can be modified to improve their mechanical and antibacterial properties. However, the included studies raise a number of important issues, such as differences in the methodology of the study, inconsistent material characterization, and the requirement for additional in vivo research to validate the in vitro findings, creating a major limitation for clinical translation. Importantly, some modification strategies showed measurable amounts of benefits such as incorporation of the bioactive glass nanoparticles, which significantly improved the compressive strength up to 0.40 to 0.45 MPa, whereas increased or excessive loading (20%) reduced the mechanical performance. Similarly, oxidized cellulose nanocrystals combined with mineral trioxide aggregates enhanced the structural stability while boron-modified bioactive glass improved the degradation resistance and also promoted odontogenic marker expression. These comparisons suggest that while mechanical strength and degradation challenges remain, targeted compositional modifications can partially overcome these limitations and thus will help in guiding future in vivo validation.

### 4.1. Critical Analysis of the Included Studies

#### 4.1.1. Biocompatibility and Cellular Response

The included studies showed the biocompatibility of cellulose-based hydrogels particularly in terms of facilitating the adhesion, proliferation, and differentiation of hDPSCs [24,25,26,27,34]. The differentiation of hDPSCs into the odontoblastic lineage was further enhanced by the inclusion of bioactive materials such as B-BG-NPs, which promoted intracellular calcium deposition and ALP activity [24]. The lack of consistent in vitro models to assess long-term cell survival and differentiation across different studies is one of the major drawbacks. Moreover, the long-term biological effects of cellulose-based hydrogels might not be fully reflected by the short-term cell culture studies of 14–21 days.

#### 4.1.2. Variability in Methodology

One of the biggest challenges to compare the findings between the included studies is the lack of standardization in the experimental methods used. Direct comparisons of the findings are very difficult since different studies synthesized and characterized cellulose-based hydrogels using different types of techniques. The use of different crosslinking agents, the various types of processing techniques used, and the testing settings led to inconsistent data on the mechanical properties, bioactivity, and degradation rates of cellulose-based hydrogels [24,25,26,27,32,33,34,35,37,39,40].

#### 4.1.3. Mechanical and Structural Limitations

Although cellulose-based hydrogels are highly biocompatible, one of the challenges is their long-term stability in oral environments is limited by their low mechanical strength, which might restrict their load-bearing capacity in dental applications. A number of studies tried to reinforce the strength of cellulose-based hydrogels by using bioactive ceramics such as boron-modified bioactive glass nanoparticles (B-BG-NPs) or hydroxyapatite (HA) in order to increase their elastic modulus and compressive [24,25]. However, it is challenging to achieve a balance between biodegradability and mechanical strength as excessive crosslinking or modifications can affect the capacity of cellulose-based hydrogels to integrate with the surrounding tissues.

#### 4.1.4. Interaction with Oral Bacteria

One of the important criteria that needs to be taken into account when using cellulose-based hydrogels is how they interact with oral bacteria. Although native cellulose-based hydrogels do not naturally include antimicrobial properties, studies indicate that functional modifications such as the incorporation of silver nanoparticles, boron-based bioactive glasses, or chitosan can significantly reduce biofilm formation and bacterial adherence [48]. Based on studies focusing on porphyromonous gingivalis and streptococcus mutants, modified cellulose-based hydrogels might help to reduce the advancements of dental caries and periodontal disease. However, the degree of antimicrobial efficacy depends on the type and concentration of the integrated antimicrobial agents [49,50].

#### 4.1.5. Biocompatibility and Degradation

The rate at which cellulose-based hydrogels degrade is largely determined by the chemical and crosslinking alterations used. According to some studies [51,52], slow degradation might prevent new tissues from integrating naturally. Other studies suggest that rapid degradation might limit their application for long-term tissue engineering applications [53,54]. Most of the cellulose-based hydrogels show excellent cytocompatibility; however, additional research is needed to determine the potential cytotoxicity of specific additives such as high boron and crosslinker glutaraldehyde.

#### 4.1.6. Cytotoxicity

The biocompatibility and cytotoxicity of cellulose-based hydrogels are important factors to consider in their application for dental tissue engineering, especially when evaluating their interaction with human dental pulp stem cells (hDPSCs). Several studies have shown that cellulose-based hydrogels generally demonstrate minimal cytotoxic effects on many cell types, including pre-osteoblasts and gingival fibroblasts; they have also shown that they promote successful cell adhesion and growth [25]. However, modifications to these hydrogels, particularly through the incorporation of bioactive nanoparticles like bioactive glass nanoparticles (BG-NPs), can affect their cytotoxic profiles. Some studies have suggested that increased concentrations of these nanoparticles may induce oxidative stress in cells, leading to a dose-dependent decrease in cell viability [55,56]. For example, Ghilan et al., 2022, indicated that carboxymethyl cellulose (CMC)-based hydrogels can be engineered with favorable mechanical and biocompatibility characteristics [56]. However, the introduction of certain agents might have a negative effect as it might impact cellular behavior [56]. Accordingly, it is crucial to carefully optimize the composition of cellulose-based hydrogels to balance their bioactivity and cytotoxicity adequately.

#### 4.1.7. Swelling Effects

Cellulose-based hydrogels exhibit swelling because of their hydrophilic properties and the existence of crosslinked polymer chains that facilitate water entrance. Excessive swelling can lead to detrimental effects, including the loss of mechanical stability and structural integrity, as well as alterations in pore morphology that may impede cell adhesion and proliferation [57]. Moreover, swelling can influence drug delivery and release; excessive swelling has been demonstrated to expedite the release of therapeutic drugs contained within the hydrogel, consequently reducing the agent’s therapeutic efficiency [58]. To tackle these issues, hydrogels must be engineered to respond to environmental cues such as pH and temperature, facilitating dynamic regulation of swelling. Consequently, attaining an adequate swelling ratio is essential for the effective performance of these hydrogels in dental applications, while preserving their desired mechanical qualities and therapeutic efficacy. The emerging literature also extends these insights towards health monitoring applications, where cellulose-based hydrogels are engineered to respond dynamically to physiological cues, enabling integration into wearable or implantable biosensing systems [13,16].

#### 4.1.8. Authors’ Perspectives, Opinion, Limitations, and Challenges

**Figure 5:**
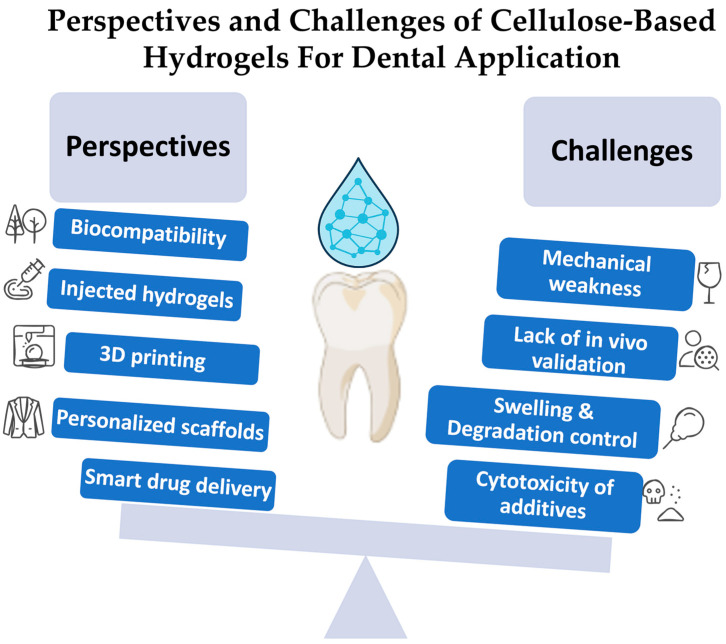
Perspectives and challenges of cellulose-based hydrogels for dental application, including mechanical stability, biodegradability, swelling behavior, and clinical translation issues.

Cellulose-based hydrogels exhibit considerable promise in dental tissue engineering; yet some essential challenges must be addressed to enable successful clinical application. A primary issue is achieving a balance between biodegradability and mechanical stability, as rapid deterioration may lead to scaffold failure, as shown in Figure 5.

Conversely, progressive degeneration may impede integration with the surrounding tissue. The enhancement in both strength and regulated degradation can be achieved through the modulation of crosslinking density. An additional issue may pertain to achieving uniform dispersion of nanoparticles, as irregular distribution could influence the mechanical properties and biological responses. Moreover, the swelling behavior has to be under control since too large an expansion can affect the drug release kinetics and scaffold orientation. Changing the cellulose content and crosslinking density helps to improve the functional stability and control of swelling. Especially for crosslinking agents and additives, cytotoxicity presents a major obstacle. Adverse tissue reactions can be avoided by means of biodegradable, non-toxic substitutes only in their development. Moreover, it is important to emphasize that in vitro results need thorough confirmation in vivo since the oral environment, such as pH changes and enzymatic activity, can influence hydrogel efficacy. More research is needed on biological factors such as hydrogels made of cellulose’s breakdown behavior and long-term biocompatibility. Comprehensive in vivo studies and clinical trials are necessary to determine the safety and effectiveness of these materials in real-world settings, even though earlier studies have produced encouraging results. Furthermore, avoiding side effects and optimizing therapeutic results will require an understanding of how cellulose-based hydrogels interact with the human immune system [59]. Environmental impact and sustainability are also becoming significant considerations in the creation of hydrogels based on cellulose. Since cellulose comes from renewable resources, it provides a more environmentally friendly option to the synthetic polymers frequently seen in dental biomaterials. In order to further minimize these materials’ environmental impact while preserving their high performance and utility, future research should concentrate on refining extraction and processing techniques [60]. Although cellulose-based hydrogel research has advanced a lot, there are several limitations that exist and need to be addressed in the future. Some of the major limitations that are essential to overcome the current knowledge gaps are that most of the current research focuses mainly on in vitro investigations and focuses on cell types, which might not be able to predict or replicate the in vivo settings accurately. Regulations are closely watched when developing biomaterials for tissue engineering when obtaining approvals from regulatory bodies such as the FDA; therefore, before the materials are used clinically, they need to undergo rigorous in vivo testing. Additionally, as the current studies mainly focus on short-term in vitro effects, the long-term patient outcomes are very difficult to predict. Thus, it is crucial to understand the long-term mechanical stability, physiological biocompatibility, enhancing the drug delivery capacity using these scaffolds, and understanding and modifying the degradation behavior and rate of cellulose-based hydrogels. Addressing these limitations will help in developing multifunctional platforms for future advancements of these materials. Filling the full potential of cellulose-based hydrogels in regenerative dentistry will depend on addressing these limitations by biomaterial innovation and thorough clinical evaluation.

## 5. Conclusions

In summary, based on the included studies, primarily in vitro (2014- 30 Nov 2024), cellulose-based hydrogels show a promising role in dentin, pulp, alveolar bone, gingival tissue regeneration, and localized drug delivery. However, translation is limited as the current evidence is almost entirely based on in vitro studies with very limited in vivo validation. Translation to clinical dentistry will require robust pre-clinical and clinical studies, along with standardized methodologies. Thus, by overcoming current limitations and promoting interdisciplinary research, cellulose-based hydrogels have the potential to revolutionize regenerative dentistry by delivering long-term and patient-specific treatment for complex dental tissue regeneration and repair. However, to ensure their clinical utility, the gap between laboratory and clinical applications must first be bridged. Future work should also explore multifunctional applications, including integration into health monitoring devices, as demonstrated in recent studies.

## Data Availability

No new data were created or analyzed in this study. Data sharing is not applicable to this article.

## Supplementary Materials

Supplementary Materials: The following supporting information can be downloaded at https://www.mdpi.com/article/10.3390/pharmaceutics17101252/s1, Supplementary Materials S1: PRISMA Checklist.

## Abbreviations

The following abbreviations are used in this manuscript:

BG-NPs	Bioactive glass nanoparticles
B	Functional ions like boron
DPSCs	Dental pulp stem cells
DSPP	Dentin sialo phosphoprotein
OPN	Osteopontin
ALP	Alkaline phosphatase
EDX	Energy-dispersive X-ray spectroscopy

## Appendix A

**Table A1:** Quantitative synthesis of compressive strength, elastic modulus, degradation loss, cell viability, porosity, and controls in the included studies.

Author (s), Year	Study Model	Compressive Strength (MPa)	Modulus (Elastic/Young/Hydrogel)	Degradation (Mass Loss %)	Cell Viability (%)	Porosity (%)	Controls (Ct) Used
**Rad et al., 2019** [24]	In vitro (hDPSCs ^1^ on CA ^2^/ox-PULL ^3^/GEL + B-BG-NPs ^4^ scaffolds)	0.40 ± 0.03	~0.5 MPa ^5^ (with BG-NPs ^6^)	~28%–37% (30 days)	120% vs Ct ^7^ (↑ ^8^ proliferation)	87.5–94.5%	Scaffolds without bioactive glass nanoparticles (BG-NPs)
**Divband et al., 2021** [26]	In vitro (injectable CMC ^9^/CSG ^10^ hydrogels with growth factor loading)	3.2–4.3 ± 0.5	3.2–5.5 MPa	Degradation ↑ with porosity	~90–100% viable (high biocompatibility)	High (interconnected)	Cells without hydrogel; base hydrogel without growth factors
**Malik et al., 2020** [32]	In vitro (thyroxine-loaded chitosan/CMC/HA hydrogels ^11^/aerogels)	Not specified	Hydrogel modulus in kPa ^12^ range	Stable (swelling-dependent)	~95–100% (cytocompatible)	Highly porous aerogels	Blank hydrogel without thyroxine; ex vivo tissue without hydrogel
**Huerta et al., 2020** [33]	In vitro (gingival fibroblasts on LL-CNF ^13^ and HL-CNF ^14^ aerogels)	Compressive strength not provided; ~264 Pa ^15^ storage modulus	~264 Pa storage modulus	Stable aerogels	~95–236% (viability upto 236% at day 11 VS control)- Highly biocompatible	Highly porous aerogels	Glass cover slips seeded with gingival fibroblasts
**Amoli et al., 2024** [34]	In vitro	Not directly given	↑ modulus with Si-HPMC ^16^	Slower degradation with Si-HPMC	~100% (good biocompatibility)	Porosity measured gravimetrically	Cells alone without microgels (no-microgel control)
**Zeeshan et al., 2018** [35]	In vitro	0.27–0.45	Modulus from stress–strain slope	7–21% (28 days)	>90% viable (pre-osteoblasts)	Porous, BG load-dependent ^17^	Scaffolds without BG (no bioactive glass)
**Pagano et al., 2019** [36]	In vitro and ex vivo	Improved with HA	Not clearly reported	Gradual degradation	↑ HDPSC proliferation and mineralization	Porous hydrogels with HA	Hydrogels without hyaluronic acid (HA)
**Lu et al., 2021** [37]	In vitro and in vivo (rats)	0.32–0.98	50–70 MPa	3–23% (28 days)	High viability in vitro and in vivo	Porosity 22–70%	Negative control: saline; positive: minocycline ointment; test: calcium phosphate cement (CPC) scaffold
**Liu et al., 2014** [38]	In vitro (Si-HPMC composite CPCs ^18^)	CPC compressive strength (wet)	Young’s modulus measured	Degradation slower with Si-HPMC	High viability (~95–100%)	23–71%	Calcium phosphate cement (CPC) alone
**Dalir et al., 2022** [27]	In vitro (injectable CS ^19^/OCNC ^20^ hydrogels with MTA ^21^)	Not specified	Hydrogel modulus reported (exact numeric value not present)	Degradation dependent on porosity	>90% viability (cytocompatible)	Highly porous injectable gels	Chitosan/oxidized cellulose nanocrystal (CS/OCNC) hydrogel without mineral trioxide aggregate (MTA); tissue culture plate
**Teti et al., 2015** [25]	In vitro	Not specified	Elastic hydrogel behavior (numeric value not specified)	Slow degradation	↑ HDPSCs viability vs. TCP ^22^	High porosity (hydrogel)	Tissue culture plastic (TCP) without scaffold
**Tritean et al., 2024** [39]	In vitro	Not reported	Hydrogel modulus (numeric value not specified)	Biodegradable	>90% viability	Porous hydrogel	Plain hydrogel without functionalization
**Srisura et al., 2024** [40]	In vitro	Not reported	Hydrogel modulus (exact numeric value not present)	Biodegradable	High viability (~95–100%)	Porous aerogels	Hydrogel without calcium phosphate (CaP) nanoparticles

1. hDPSCs—human dental pulp stem cells; 2. CA—cellulose acetate; 3. ox-PULL—oxidized pullulan–gelatin; 4. B-BG-NPs—boron-modified bioactive glass nanoparticles; 5. MPa—megapascal; 6. BG-NPS—boron-modified bioactive glass nanoparticles; 7. Ct—control; 8. ↑—increased; 9. CMC—carboxymethyl cellulose; 10. CSG—chondroitin sulfate graft; 11. CMC/HA hydrogels—carboxymethyl cellulose hyaluronic acid hydrogels; 12. Kpa—kilopascal; 13. LL-CNF—low-lignin cellulose nanofibril aerogel; 14. HL-CNF—high-lignin cellulose nanofibril aerogel; 15. Pa—pascal; 16. Si-HPMC—silanized hydroxypropyl methylcellulose (polymer additive); 17. BG—bioactive glass; 18. CPCs—calcium phosphate cement; 19. CS—chitosan; 20. OCNC—oxidized cellulose nanocrystal; 21. MTA—mineral trioxide aggregate; 22. TCP—Tissue culture plastic.

**Table A2:** Minimal quality assessment table of the included studies.

Study (Authors, Year)	Model System (Cell Line/Primary/In Vivo/Others)	Sample Size (Per Assay as Reported)	Controls Used (Name)
**Rad et al., 2019** [24]	Primary hDPSCs ^1^, in vitro	Cell assays in triplicate (*n* = 3); ALP ^2^/calcium *n* = 4	Scaffolds without BG-NPs ^3^; medium-only
**Divband et al., 2021** [26]	Primary hDPSCs, in vitro	MTT ^4^/viability in triplicate (*n* = 3)	Untreated cells (cells + medium)
**Malik et al., 2020** [32]	MC3T3 ^5^ cells in vitro + CAM assay (ex vivo)	CAM ^6^ bead counts *n* = 3; in vitro triplicates	Control hydrogel (no thyroxine); untreated wells
**Huerta et al., 2020** [33]	Primary oral/gingival cells, in vitro	Assays performed in duplicate	Blank glass/untreated scaffolds
**Amoli et al., 2024** [34]	Primary hDPSCs, in vitro	DNA ^7^ and viability assays in triplicate (*n* = 3)	Control microgel formulations; medium-only
**Zeeshan et al., 2018** [35]	MC3T3-E1 cell line, in vitro	Cell assays in triplicates	Tissue culture plastic (TCP ^8^); untreated cells
**Pagano et al., 2019** [36]	In vitro release + ex vivo porcine buccal mucosa	Gelling/release/mucoadhesion *n* = 3	Blank formulations; porcine mucosa baseline
**Lu et al., 2021** [37]	In vitro hDPSCs + in vivo rat periodontal defect model	Animal groups *n* = 3–6; in vitro triplicates	Saline control; minocycline ointment
**Liu et al., 2014** [38]	In vitro (cement materials only)	Setting times mean of 3; mechanical properties mean of 6	Control CPC ^9^ (no Si-HPMC ^10^)
**Dalir et al., 2022** [27]	Primary hDPSCs on cellulose scaffold, in vitro	Cell assays (MTT, ALP, ARS ^11^) carried out in triplicate (*n* = 3)	Untreated HDPSCs (medium only); blank scaffolds without bioactive glass particles
**Teti et al., 2015** [25]	Primary hDPSCs, in vitro	Proliferation, viability, ALP assays performed in triplicate (*n* = 3)	Negative control: cells cultured on tissue culture plastic (TCP); scaffold without hydroxyapatite additive
**Tritean et al., 2024** [39]	Primary hDPSCs, in vitro	qRT-PCR ^12^, viability, mineralization assays all reported in triplicate (*n* = 3)	Blank scaffold without active components; untreated cells
**Srisura et al., 2024** [40]	Primary hDPSCs, in vitro	Cytotoxicity/viability assays/ qRTPCR/Western blot, ALP assay, Alizarin red assay all conducted in triplicate (*n* = 3)	Multiple controls: (1) cells alone without hydrogels (negative control); (2) GAPDH ^13^ as house-keeping control in qRT-PCR; (3) β-actin ^14^ as loading control in Western blot.

1. hDPSCs—human dental pulp stem cells; 2. ALP—alkaline phosphatase; 3. BG-NPs—boron-modified bioactive glass nanoparticles; 4. MTT—3-(4, 5-dimethylthiazol-2-yl)-2,5-diphenyltetrazolium bromide; 5. MC3T3-E1—mouse osteoblast cell line; 6. CAM—chick chorioallantoic membrane (ex ovo angiogenesis assay in chicken embryos); 7. DNA—deoxyribonucleic acid; 8. TCP—tissue culture plastic; 9. CPC—calcium phosphate cement; 10. Si-HPMC—silanized hydroxypropyl methylcellulose (polymer additive); 11. ARS—Alizarin Red S (staining for calcium deposits/mineralization); 12. qRT-PCR—quantitative real-time polymerize chain reaction; 13. GAPDH—Glyceraldehyde-3-phosphate Dehydrogenase (house-keeping gene); 14. β-actin—beta-actin.
